# Supplementary material for: Multispectral optoacoustic imaging of dynamic redox correlation and pathophysiological progression utilizing upconversion nanoprobes
Source: Nat Commun. 2019 Mar 6;10:1087. doi: 10.1038/s41467-019-09001-7 (PMC6403272; doi:10.1038/s41467-019-09001-7)
Supplement: Supplementary file 1 — Supplementary Information [file 41467_2019_9001_MOESM1_ESM.pdf]

Supplementary Information

Multispectral Optoacoustic Imaging of Dynamic Redox Correlation and Pathophysiological Progression Utilizing Upconversion Nanoprobes

Xing et al.

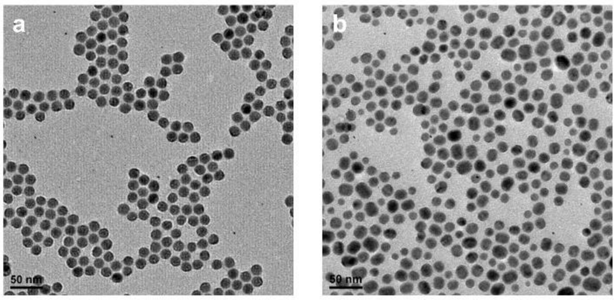

Supplementary Fig. 1. TEM images of the as-prepared UCNs nanostructures. (a) $NaGdF_4$: Yb/Er/Tm (20/2/1%) core UCNs; (b) $NaGdF_4$: Yb/Er/Tm (20/2/1%) @ $NaGdF_4$ core-shell UCNs. Scale bar: 50 nm.

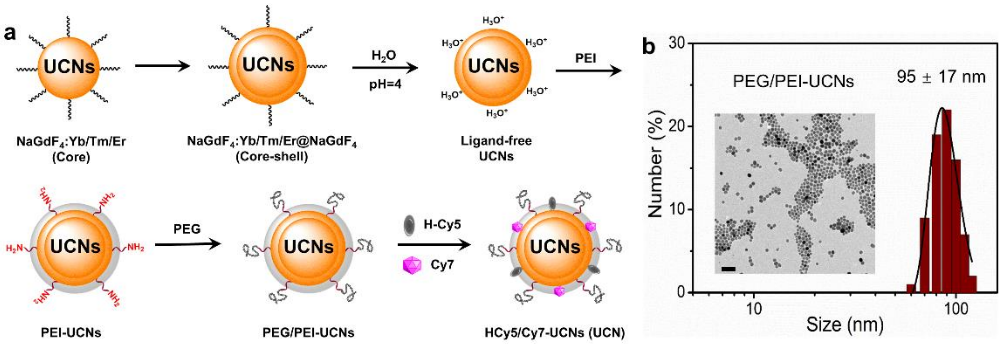

Supplementary Fig. 2. (a) Design and synthesis of ROS/RNS-sensitive photon-upconverting nanoprobe (UCN). (b) TEM and DLS analysis of PEG/PEI-UCNs. Scale bar: 100 nm.

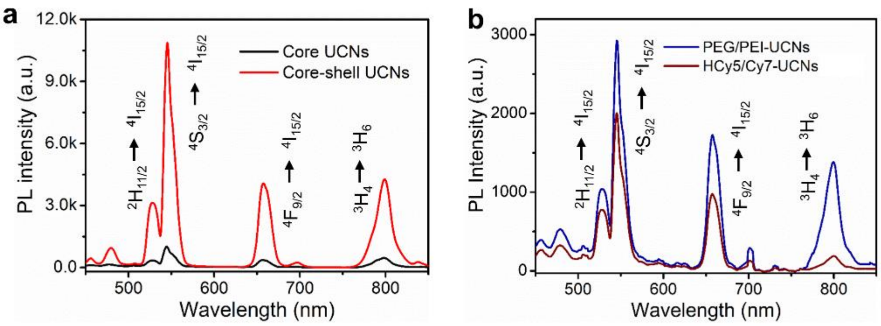

Supplementary Fig. 3. The upconverted luminescence (UCL) spectra of (a) core and core-shell UCNs; (**b**) PEG/PEI-UCNs and HCy5/Cy7-UCNs (UCN) upon 980 nm NIR light excitation. The concentration of all nanoparticle samples is 1 mg mL^{-1}.

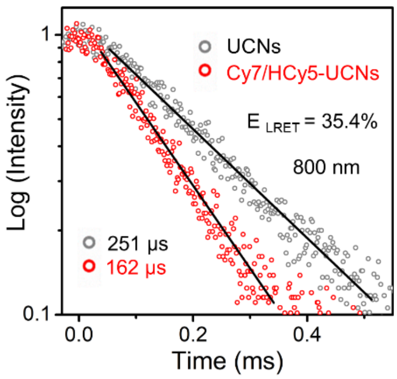

Supplementary Fig. 4. The upconverted luminescence (UCL) lifetime decay curves of UCNs nanoparticles at 800 nm with (red) or without (gray) surface encapsulation of Cy7 (E_x: 980 nm). The LRET efficiency (E_{LRET}) = 1 - τ_{DA}/ τ_D, where τ_{DA} and τ_D are the lifetime of UCNs at 800 nm in the absence (gray) and presence (red) of fluorophore (Cy7) loading, respectively.

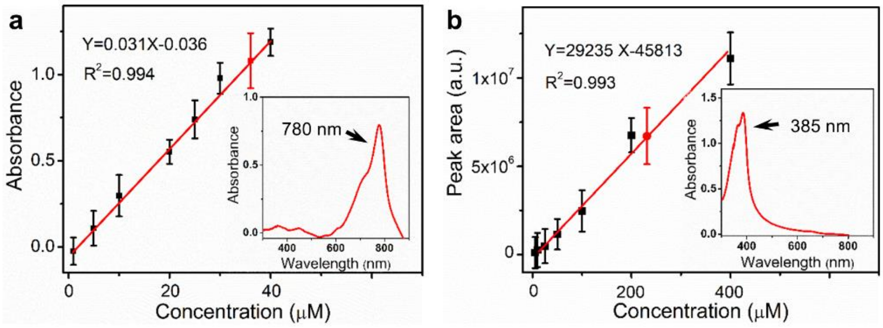

Supplementary Fig. 5. Quantification of Cy7 and HCy5 on UCNs nanoprobe. (a) The standard curve for Cy7 based on its absorbance at 780 nm. (b) The standard curve for HCy5 based on its absorbance at 385 nm. HPLC was applied to quantify the amounts of fluorophores on UCNs.[1, 2]

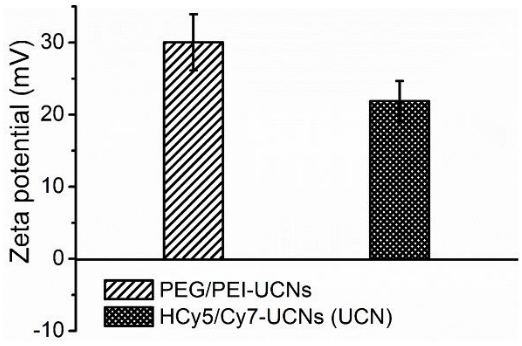

Supplementary Fig. 6. Zeta potential analysis of PEG/PEI-UCNs and HCy5/Cy7-UCNs (UCN) in PBS (pH = 7.4).

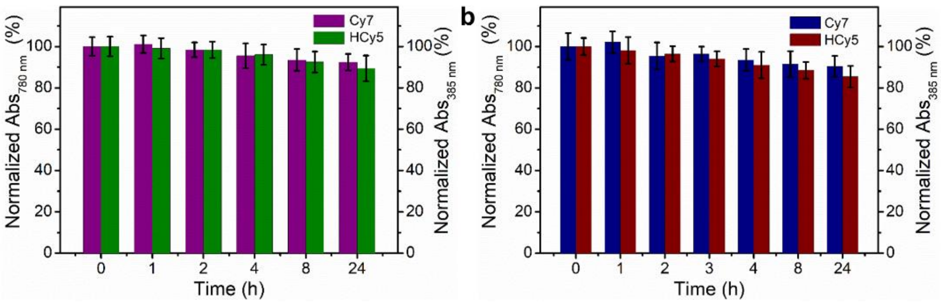

Supplementary Fig. 7. Stability test of loaded Cy7 and HCy5 molecules on the surface of UCNs nanoprobe in DMEM (**a**) and mouse serum (**b**) after incubation at different time based on their specific absorbance at 780 nm and 385 nm respectively. Data were represented as mean ± SD.

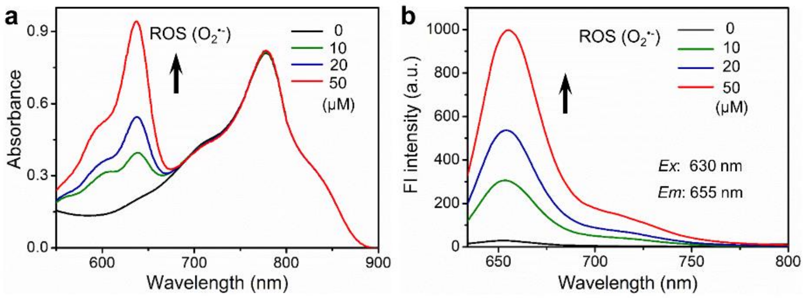

Supplementary Fig. 8. The UV-vis spectra (a) and fluorescence emission (b) of UCN (1 mg mL^{-1}) upon different concentration of ROS (*e.g.*, superoxide anion, $O_2^{\bullet-}$) treatment.

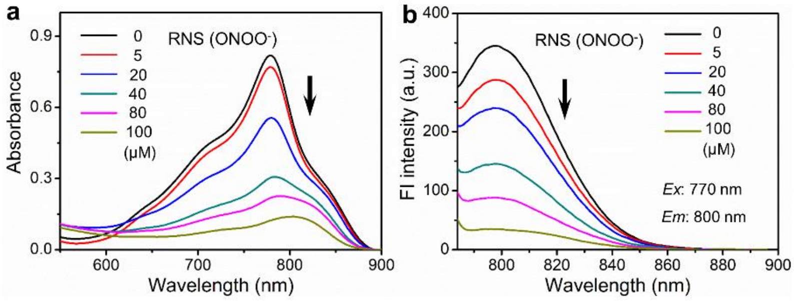

Supplementary Fig. 9. The UV-vis spectra (a) and luminescence emission (b) of UCN (1 mg mL^{-1}) upon different concentration of RNS (*e.g.,* peroxynitrite, $ONOO^-$) treatment.

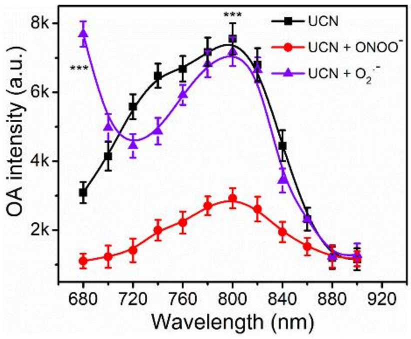

Supplementary Fig. 10. OA spectra of UCN in the absence and presence of $O_2^{\cdot-}$ and ONOO$^-$ (100 μM) treatment. Data were represented as mean ± SD.

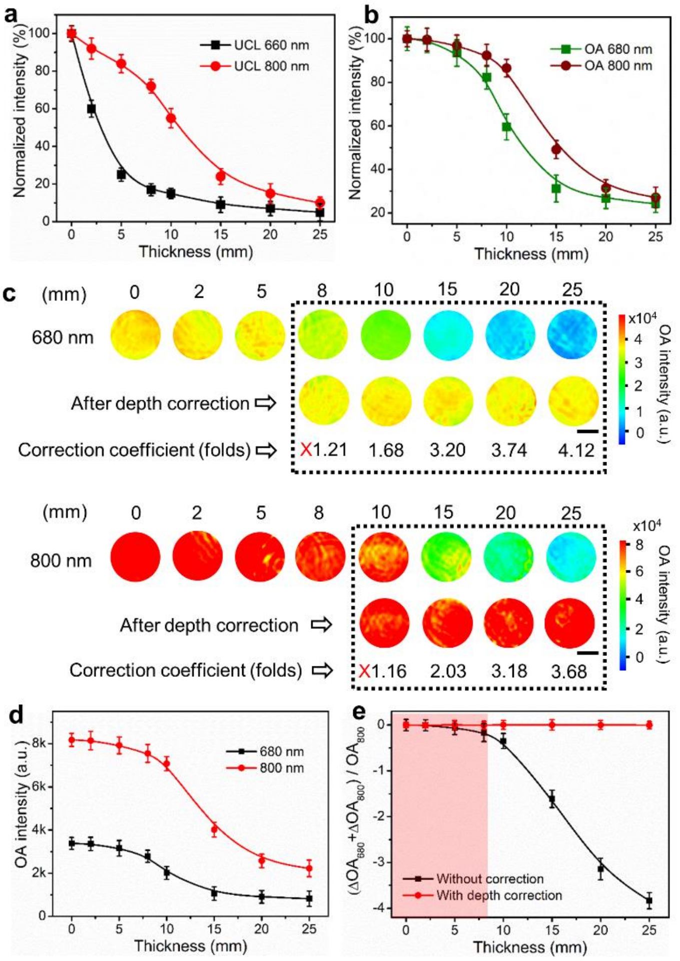

Supplementary Fig. 11. The penetration depth examination of UCL and OA imaging of UCN (1 mg mL^{-1}) with pork tissues at different thickness. (a) Normalized UCL signals at 660 and 800 nm. **(b)** Normalized OA signals at 680 and 800 nm. **(c)** MSOT phantom images of UCN at 680 and 800 nm with or without penetration depth calibration. Scale bar: 1 mm. **(d, e)** Absolute OA signals (**d**) and ratiometric values (**e**) of UNC at 680 nm and 800 nm at different tissue thickness. Data were represented as mean ± SD.

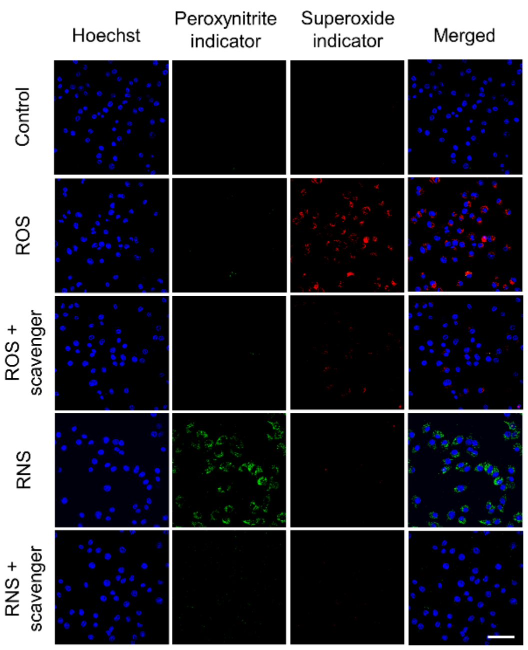

Supplementary Fig. 12. Confocal imaging of ROS and RNS generation in RAW264.7 cells upon the traetment of PMA and LPS/INF-γ/PMA followed by MnTBAP and MEG as specific scavengers for superoxide ($O_2^{\bullet -}$) and peroxynitrite ($ONOO^-$) species, respectively. Blue: Hoechst 33342 (E_x: 405 nm, E_m: 460/50 nm), green: peroxynitrite indicator (E_x: 480 nm, E_m: 520/50 nm), red: superoxide indicator (E_x: 565 nm, E_m: 590/40 nm). Scale bar: 50 μm.

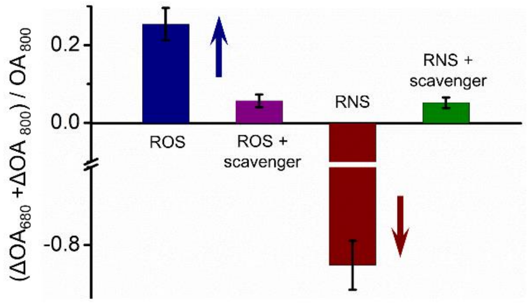

Supplementary Fig. 13. The reversed ratiometric OA signals of UCN at 680 nm and 800 nm incubated with RAW264.7 cells upon treatment with PMA ($O_2^{\bullet -}$ generation), LPS/ INF-γ/PMA ($ONOO^-$ generation), and their specific scavenger (MnTBAP for $O_2^{\bullet -}$ and MEG for $ONOO^-$), respectively. Data were represented as mean ± SD.

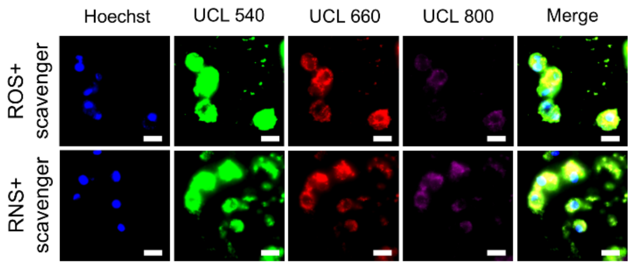

Supplementary Fig. 14. UCL fluorescence imaging of RAW264.7 cells incubated with UCN (100 μg mL^{-1}) upon treatment with PMA and LPS/ INF-γ/PMA followed by specific scavengers of $O_2^{\bullet-}$ (top) and $ONOO^-$ (bottom), respectively. Blue: Hoechst 33342 (E_x: 405 nm, E_m: 460/50 nm), green: UCL-540 (E_x: 980 nm, E_m: 540/50 nm), red: UCL-660 (E_x: 980 nm, E_m: 640/50 nm), violet: UCL-800 (E_x: 405 nm, E_m: 790/30 nm). Scale bar: 20 μm.

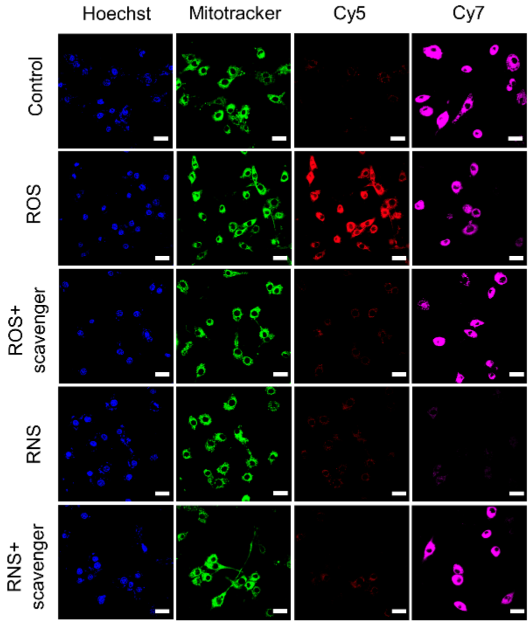

Supplementary Fig. 15. Confocal imaging of RAW264.7 cells incubated with UCN (100 μg mL^{-1}) upon treatment with PMA and LPS/ INF-γ/PMA followed by specific scavenger of $O_2^{\bullet-}$ (top) and $ONOO^-$ (bottom), respectively. Blue: Hoechst 33342 (E_x: 405 nm, E_m: 460/50 nm), green: Mitochondria tracker (E_x: 480 nm, E_m: 520/50 nm), red: Cy5 (E_x: 590 nm, E_m: 630/50 nm), violet: Cy7 (E_x: 780 nm, E_m: 790/30 nm). Scale bar: 20 μm.

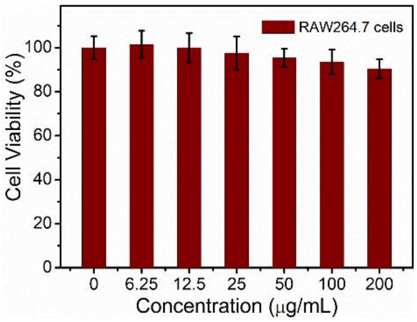

Supplementary Fig. 16. Cell viability test of RAW264.7 cells treated with UCNs at various concentrations for 24 h. Data were represented as mean ± SD.

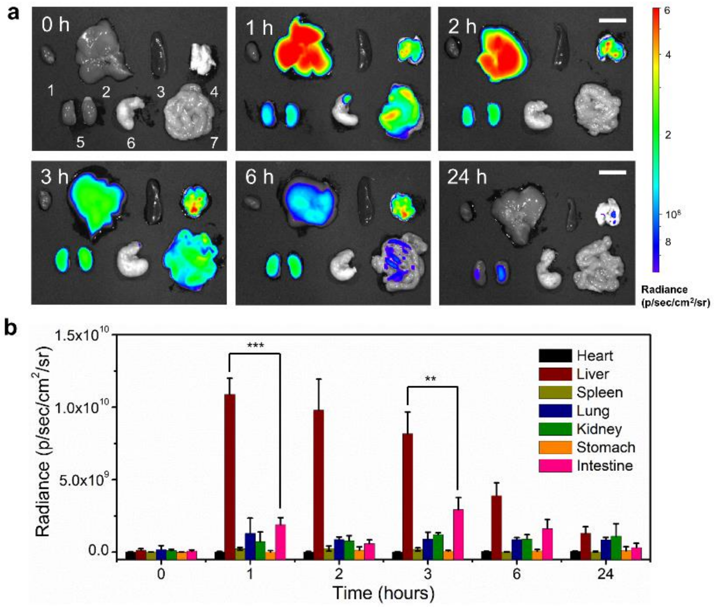

Supplementary Fig. 17. Bio-distribution of UCN in various organs of mice (a) and quantification of their UCL signals at 800 nm (b) after tail-vein injection in different time. The UCL signals of UCN at 800 nm were recorded upon 980 nm NIR light excitation (E_m: 790/30 nm). The organs from 1-7: 1, Heart; 2, Liver; 3, Spleen; 4, Lung; 5, Kidney; 6, Stomach; 7, Intestine. Scale bar: 1 cm. Statistical significance assessed by a Student's t-test (heteroscedastic, two-sided). ***$p < 0.001$; **$p < 0.01$. Data were represented as mean ± SD.

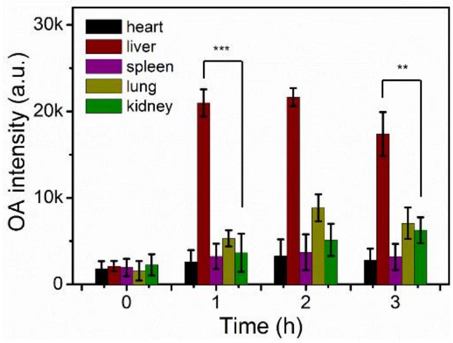

Supplementary Fig. 18. Quantification of OA signals at 800 nm in the homogenates of various organs after UCN injection at different time. Statistical significance assessed by a Student's t-test (heteroscedastic, two-sided). ***$p < 0.001$; **$p < 0.01$. Data were represented as mean ± SD.

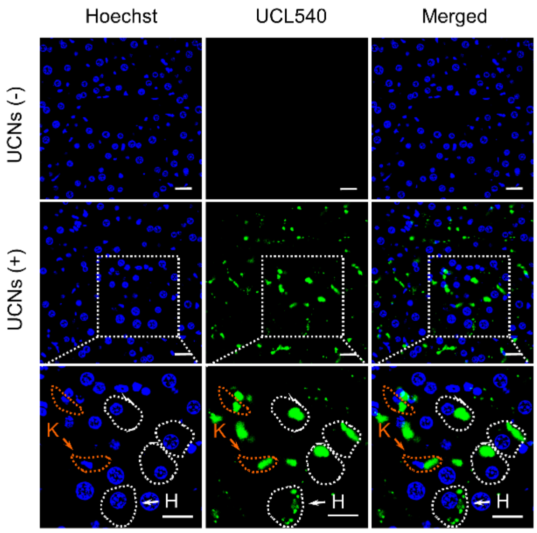

Supplementary Fig. 19. Fluorescence analysis of the UCN nanoprobe uptake and biodistribution in mouse liver tissues. Blue: Hoechst 33342 (E_x: 405 nm, E_m: 460/50 nm); Green: UCL540 (E_x: 980 nm, E_m: 540/50 nm); K: Kupffer cells (red); H: hepatocytes (white). Scale bar: 20 μm.

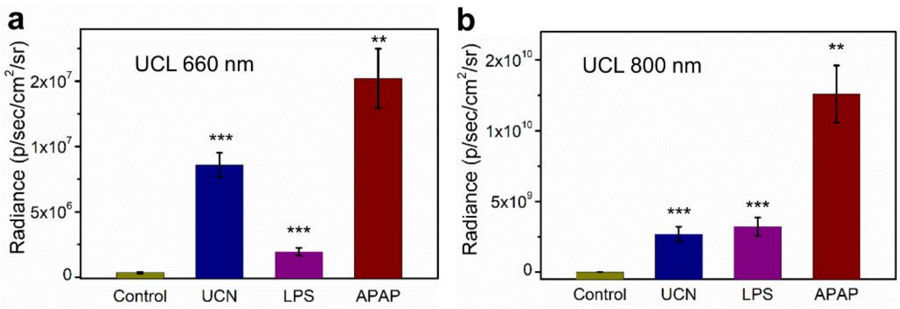

Supplementary Fig. 20. Quantification of the UCL signals at 660 nm (a) and 800 nm (b) in mice liver at 120 min in the absence and presence of UCN, LPS and APAP administration (*n* = 5). Data were represented as mean ± SD.

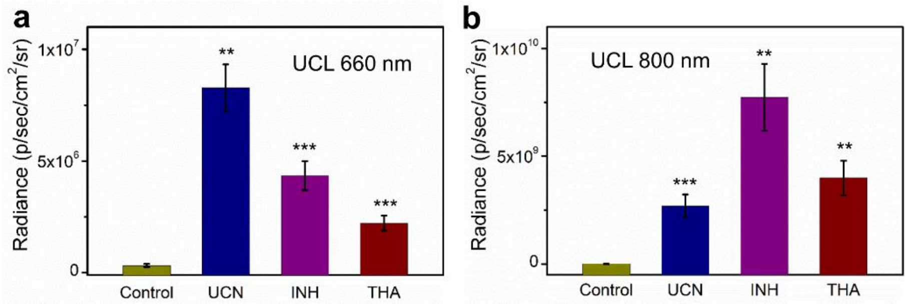

Supplementary Fig. 21. (a) Quantification of the UCL signals at 660 nm in mice liver before and after the treatment of UCNs (at 30 min), INH (at 30 min), and THA (at 60 min) respectively (*n* = 5). **(b) Quantification of the UCL signals at 800 nm in mice liver** before and after the stimulation of UCNs, INH and THA for 120 min respectively (*n* = 5). Statistical significance assessed by a Student's t-test (heteroscedastic, two-sided). ***$p < 0.001$; **$p < 0.01$. Data were represented as mean ± SD.

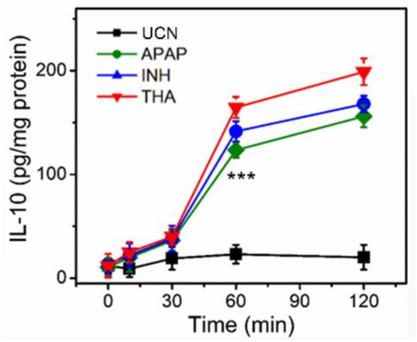

Supplementary Fig. 22. Variations of inflammation-associated cytokine (IL-10) upon various hepatotoxic drugs administration at different time in liming mice (n = 5). Data were represented as mean ± SD.

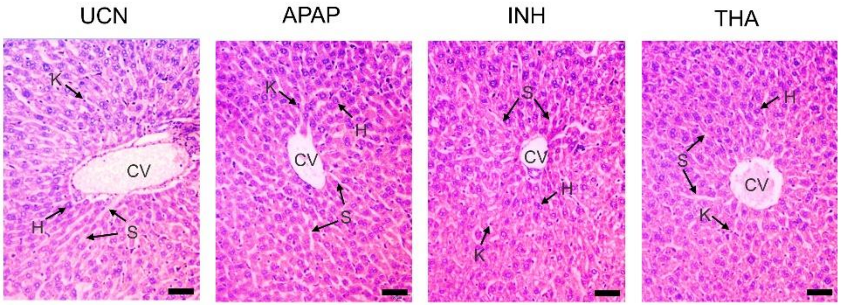

Supplementary Fig. 23. H&E staining in liver tissues at 60 min upon UCNs, APAP, INH and THA treatment (n = 5). Arrows mark the typically histological structures of liver tissues. S: sinusoid; H: hepatocyte; K: Kupffer cell; CV: central vein. Scale bars: 50 μm.

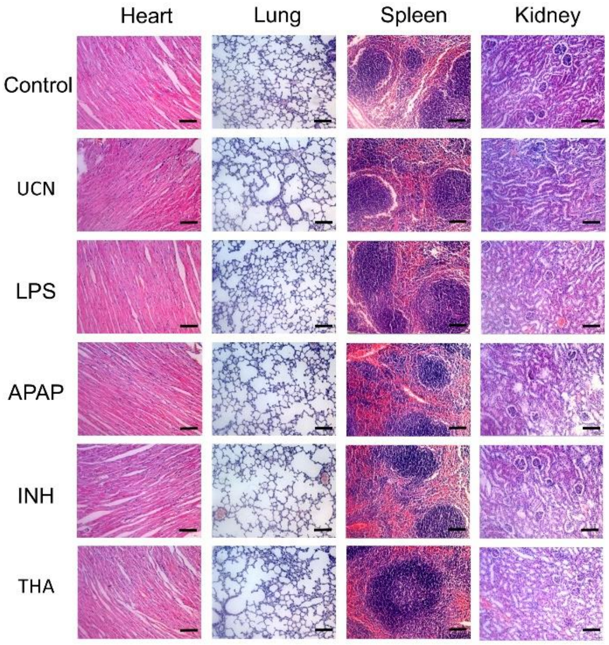

Supplementary Fig. 24. H&E staining of various organs upon UCN, LPS, APAP, INH and THA treatment (n = 5). The organs (*e.g.,* heart, lung, spleen, kidney) were harvested at 180 min after drugs treatment. Scale bars: 100 μm.

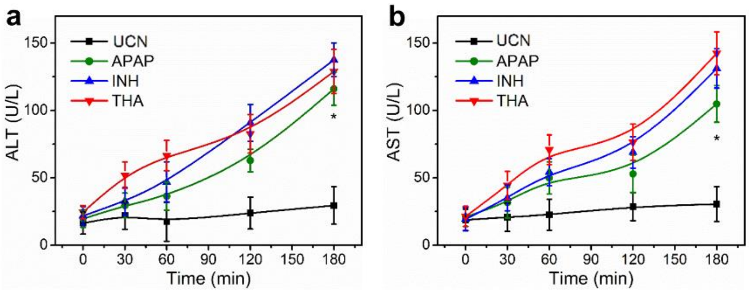

Supplementary Fig. 25. Quantification of ALT (a) and AST (b) biomarkers variations at different time upon different drugs administration (n = 5). Data were represented as mean ± SD.

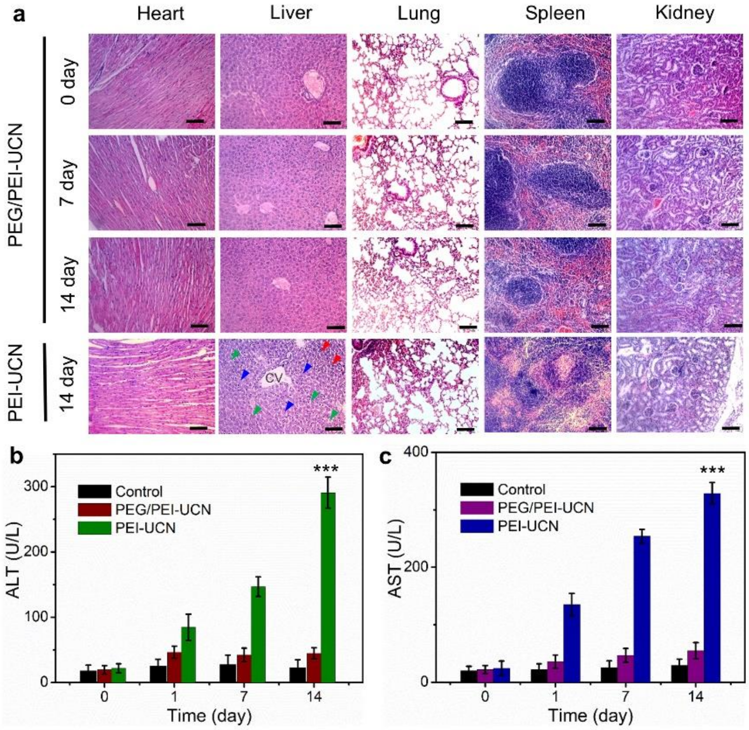

Supplementary Fig. 26. Investigation of the longer-term toxicity of UCNs nanoplatform through histological and hepatoxic biomarkers analysis. (**a**) H&E staining of various organs upon PEG/PEI-UCN and PEI-UCN treatment including heart, lung, spleen and kidney. These organs were harvested at 0, 7 and 14 days after nanoparticle *i.v.* injection in living mice. Arrowheads mark centrilobular vein fibrosis (blue), swollen hepatocytes (green) and inflammatory infiltration (red) respectively. CV: central vein. Scale bars: 200 μm. (**b, c**) Quantification of ALT (**b**) and AST (**c**) biomarkers variations in mice liver at different days upon PEG/PEI-UCN and PEI-UCN administration (n = 5). Data were represented as mean ± SD.

Supplementary Methods

Chemicals and reagents. $Gd(CH_3CO_2)_3$, $Yb(CH_3CO_2)_3$, $Er(CH_3CO_2)_3$, $Tm(CH_3CO_2)_3$, oleic acid, 1-octadecene, NH_4F, NaOH, polyacrylic acid (PAA, Mw 1800), diethylene glycol (DEG), polyethylenimine (PEI, Mw 25000, branched), *In vitro* toxicology assay kit (TOX8, resazurin based), and bisBenzimide H 33342 trihydrochloride (Hoechst) were purchased from Sigma-Aldrich. The MitoSOX mitochondrial superoxide indicator (red fluorescence) and Cell Meter™ fluorimetric intracellular peroxynitrite assay kit (green fluorescence), anti-3-nitrotyrosine and anti-4-Hydroxynonenal antibody were bought from Abcam (USA). Dulbecco's Modified Eagle Medium (DMEM), fetal bovine serum (FBS), penicillin-streptomycin and trypsin-EDTA were obtained from Invitrogen (USA). All the commercially reagents were used as received unless otherwise noted.

Instruments. ^{1}H-NMR and ^{13}C-NMR spectra were measured on a 300 MHz Bruker spectrometer. Mass spectra were measured on a Thermo Polaris Q instrument for EI measurements and a Thermo LCQ Deca XP MAX instrument for ESI measurements. High performance liquid chromatography (HPLC, Shimadzu) system was performed on an Alltima C-18 column of 250×10 mm at a flow rate of 3 mL min^{-1}. UV-vis absorption spectra were measured using a Beckman coulter DU800 spectrometer. Fluorescence emission spectra were captured with an Agilent Varian Cary eclipse fluorescence spectrophotometer. The fluorescence emission spectra of UCNs were recorded at an angle of 90° to the excitation laser (980 nm, 2 W cm^{-2}) and an optical SEC-2000 spectrometer coupled 2048 pixels CCD assay (ALS Co., Ltd, Japan). Transmission electron microscope (TEM) images were recorded using a FEI EM208S TEM (Philips) operated at 100 kV. Dynamic light scattering (DLS) and zeta potential measurements were performed by Brookhaven 90 Plus nanoparticle size analyzer. The cell viabilities were measured by a Bio-Tek EL-311 microplate reader. The confocal imaging of cells was carried out on Carl Zeiss LSM 800 confocal laser microscope (Germany). The upconversion luminescence (UCL) and Cy7 emission in living cells were recorded separately on a Nikon confocal fluorescence microscope (Nikon, Eclipes TE2000-E, Japan) equipped with 980 nm and 786 nm laser wide-field fluorescence add-on (EINST Technology Pte Ltd, Singapore) in our lab. The luminescence lifetime decay curves were measured with a phosphorescence lifetime spectrometer (FSP920, Edinburgh) equipped with a microsecond flash lamp as the excitation source. All the optoacoustic (OA) imaging experiments *in vitro* and *in vivo* were performed by using a real-time multispectral optoacoustic tomographic (MSOT) imaging system from iThera Medical GmbH (Neuherberg, Germany). The UCL imaging in living mice was performed with an IVIS Lumina II imaging system (Caliper Life Sciences, France) with 980 nm NIR laser irradiation.

1. Preparation of radical species responsive fluorophores

1.1) Synthesis of RNS-responsive molecule Cy7.

The RNS-sensitive Cy7 probe was synthesized by following a reported method (Supplementary Fig. 27).[3] Typically, the starting material **IR780** (183 mg, 0.28 mmol), 3-mercaptopropionic acid (28 μL, 0.32 mmol) and triethylamine (TEA, 44 μL, 0.32 mmol) were mixed in 6.0 mL dimethylformamide (DMF) and stirred at room temperature for 24 h. Then the mixture solution was added drop-wisely into ice-cold methyl ether and the crude product as green precipitate was filtrated. The final product was purified by flash chromatography with the gradient of CH_2Cl_2/CH_3OH (changed from 100: 1 to 10: 1) to afford **Cy7** as a powder (yield: 169.3 mg, 75%). ^{1}H NMR (300 MHz, MeOD): δ 8.90 (d, J = 14.2 Hz, 2H), 7.53 (d,

J = 7.4 Hz, 2H), 7.47-7.39 (m, 2H), 7.30 (dd, J = 15.6, 7.8 Hz, 4H), 6.32 (d, J = 14.2 Hz, 2H), 4.15 (t, J = 7.2 Hz, 4H), 3.33 (dt, J = 3.3, 1.6 Hz, 4H), 3.09 (t, J = 6.9 Hz, 2H), 2.70 (s, 2H), 2.61 (t, J = 6.9 Hz, 2H), 1.91 (dd, J = 14.7, 7.4 Hz, 4H), 1.78 (s, 12H), 1.08 (t, J = 7.4 Hz, 6H). ^{13}C NMR (75 MHz, MeOD) δ 173.80, 172.73, 145.79, 142.48, 141.07, 134.81, 128.39, 124.83, 122.07, 110.67, 100.69, 49.13, 45.17, 34.70, 32.75, 26.99, 25.84, 20.79, 20.43, 14.00, 10.28. ESI-MS: $[M-I]^+$ m/z: 609.59.

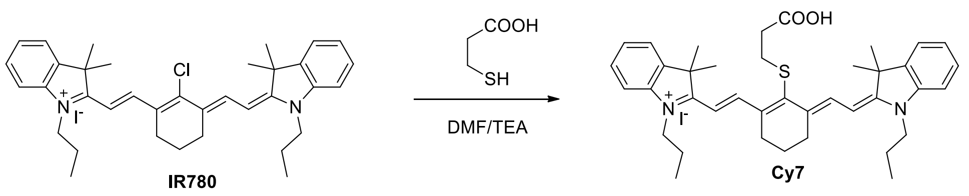

Supplementary Fig. 27. Synthesis of RNS-responsive molecule Cy7.

1.2) Synthesis of ROS-responsive molecule HCy5.

Typically, three steps were required to synthesize HCy5 by following a reference published before,[4] as shown in Supplementary Fig. 28.

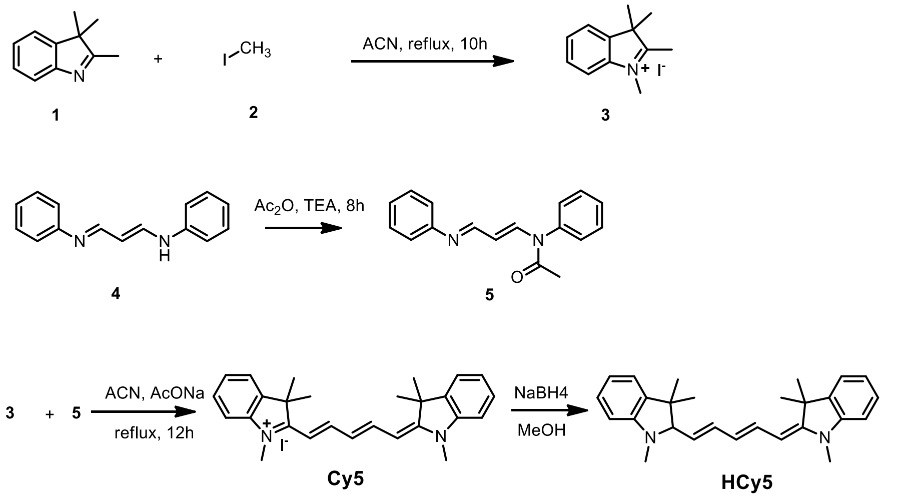

Supplementary Fig. 28. Synthesis of ROS-responsive molecule HCy5.

1.2.1) Synthesis of compound 3.

The compounds 2,3,3-trimethylindolenine (**1,** 1 ml, 6.2 mmol) and methyl iodide (**2**, 0.9 ml, 12.5 mmol) were dissolved in 20 ml acetonitrile (ACN). The mixture solution was refluxed for 10 h, then allowed to cool down to room temperature, and the precipitate was collected by filtration. The solid was washed with *n*-hexane and dried under reduced pressure to afford compound **3** as a brown solid. Yield: 1.7 g (85%). ^{1}H NMR (300 MHz, $CDCl_3$): δ 7.61 (d, J = 13.5, 8.0, 3.6 Hz, 4H), 4.29 (s, 3H), 3.13 (s, 3H), 1.59 (s, 6H). ESI-MS: $[M-I]^+$ m/z: 232.30.

1.2.2) Synthesis of Cy5.

Malonaldehyde dianilide hydrochloride (**4**, 400 mg, 1.55 mmol) was dissolved in a mixture of dichloromethane (DCM, 10 mL) and TEA (0.6 mL), and then added drop-wisely to the solution of acetic anhydride (Ac_2O, 0.4 mL) in DCM (3 mL) followed by vigorous stirring at room temperature for 8 h. The solid product was obtained by evaporating the mixture without further purification, and then compound **5** (130 mg, 0.5 mmol) was dissolved in 1 mL ACN solution followed by drop-wisely adding

in a refluxing ACN solution (9 mL) containing compound **3** (300 mg, 0.1 mmol) and sodium acetate (50 mg, 0.78 mmol). After 12 h refluxing, the reaction mixture was allowed to cool down at room temperature, and the precipitate was removed by filtration. The crude product was further purified by flash chromatography with the gradient of DCM/CH_3OH (changed from 100: 1 to 10: 1) to afford **Cy5** as a powder (yield: 114 mg, 45%). ^1H NMR (300 MHz, $CDCl_3$): δ 7.95 (t, J = 13.1 Hz, 2H), 7.50-7.32 (m, 4H), 7.25 (d, J = 7.6 Hz, 2H), 7.13 (d, J = 7.8 Hz, 2H), 6.89 (t, J = 12.7 Hz, 1H), 6.35 (d, J = 13.6 Hz, 2H), 3.70 (s, 6H), 1.76 (s, 12H). ^{13}C NMR (75 MHz, $CDCl_3$): δ 173.37, 152.81, 142.40, 140.73, 128.42, 126.52, 125.40, 122.13, 110.33, 104.04, 54.61, 31.97, 28.14. ESI-MS: $[M-I]^+$ *m/z*: 383.43. HRMS (ESI): $[M-I]^+$ *m/z*: 383.2491 (calcd. for 383.2482, $C_{27}H_{31}N_2$).

1.2.3) Synthesis of HCy5.

The prepared **Cy5** (52 mg, 0.1 mmol) was dissolved in 5 mL methanol, then another 0.5 mL methanol containing $NaBH_4$ (3.0 mg, 0.08 mmol) was added drop-wisely to the **Cy5** solution and stirred for 10 min until the solution changed to colorless. The reaction mixture was further magnetically stirred for 20 min and removed the solvent under reduced pressure. The residue was dissolved in 10 mL DCM, and extracted by 5 mL water with vigorously shaken. The collected organic layer was dried by anhydrous Na_2SO_4, and the solvent was finally removed under reduced pressure. The product **HCy5** thus was obtained without further purification. ^1H NMR (300 MHz, $CDCl_3$) δ 7.17 (dd, J = 15.7, 7.5 Hz, 3H), 7.04 (t, J = 11.8 Hz, 1H), 6.92-6.73 (m, 3H), 6.59 (dd, J = 12.3, 7.9 Hz, 2H), 6.46 (dd, J = 14.8, 10.8 Hz, 1H), 6.17 (dd, J = 14.0, 11.1 Hz, 1H), 5.62 (dd, J = 14.9, 9.1 Hz, 1H), 5.34 (d, J = 11.4 Hz, 1H), 3.33 (d, J = 9.1 Hz, 1H), 3.13 (s, 3H), 2.71 (s, 3H), 1.63 (s, 6H), 1.34 (s, 3H), 1.10 (s, 3H). ^{13}C NMR (75 MHz, $CDCl_3$) δ 156.58, 151.64, 145.38, 139.40, 138.74, 136.15, 129.29, 127.69, 127.45, 125.66, 124.44, 121.67, 121.50, 119.10, 118.38, 107.88, 105.57, 95.83, 81.07, 45.26, 44.29, 34.36, 28.99, 28.34, 28.31, 25.62, 24.12. ESI-MS: $[M+H]^+$ *m/z*: 385.47. HRMS (ESI): $[M+H]^+$ *m/z*: 385.2647 (calcd. for 385.2644, $C_{27}H_{33}N_2$).

2. Preparation of HCy5-Cy7 modified upconversion nanoplatform (UCN)

2.1) Synthesis of $NaGdF_4$:Yb/Er/Tm (20/2/1%) core upconversion nanocrystals (UCNs):

The core UCNs were obtained following a reference published before.[5] Typically, 2 mL methanol containing $RE(CH_3CO_2)_3$ (RE = Gd, Yb, Er and Tm), 3 mL oleic acid and 7 mL 1-octadecene were added in a 50 mL three-neck flask. The ratio of Yb/Er/Tm is 20/2/1% and the total lanthanide amount is 0.4 mmol. The mixture was heated to 150 °C for 60 min before cooling down to room temperature. Subsequently, a methanol solution (6 mL) containing NH_4F (59.3 mg) and NaOH (40.0 mg) was added and stirred for 30 min at 50 °C. Then methanol was evaporated and the solution was kept at 290 °C for 1.5 h under nitrogen atmosphere. The core particles were then precipitated by ethanol after cooling down to room temperature, collected through centrifugation several times after ethanol washing, and re-dispersed in 4 mL hexane for next step.

2.2) Synthesis of $NaGdF_4$:Yb/Er/Tm (20/2/1%)@$NaGdF_4$ core-shell UCNs:

Typically, 3 mL oleic acid and 7 mL 1-octadecene were added in a 50 mL three-neck flask, then 2 mL methanol containing 0.4 mmol $Gd(CH_3CO_2)_3$ was also added in the solution. The mixture was heated to 150 °C for 60 min before cooling down to room temperature. The as-synthesized $NaGdF_4$:Yb/Er/Tm core particles in 4 mL hexane were added along with a methanol solution (6 mL) containing NH_4F (59.3

mg) and NaOH (40.0 mg). The resulting mixture was stirred for 30 min at 50 °C. Then methanol was evaporated and the solution was kept at 290 °C for 1.5 h under nitrogen atmosphere. After cooling down to room temperature, the nanoparticles were precipitated using ethanol and collected through centrifugation several times after ethanol washing. Finally, the core-shell UCNs were re-dispersed in 4 mL cyclohexane and conserved in 4 °C fridge.

2.3) Synthesis of PEI-UCNs:

The PEI-UCNs synthesis was carried out following a literature protocol reported previously.[6] Briefly, the as-prepared oleate-capped UCNs were precipitated using ethanol and re-dispersed in a 10 mL acid aqueous solution (pH = 4) adjusted by HCl (0.1 M). Then the solution was sonicated for 30 min following with vigorous stirring for 2 h. During this reaction the carboxylate groups of the oleate ligands were protonated (to yield oleic acid). Then the aqueous solution was extracted with 30 mL diethyl ether to remove the oleic acid for three times. The combined ether layers were re-extracted with 10 mL water. Finally, the dispersible ligand-free UCNs in the water were recuperated by centrifugation (44,720 ×g, 10 min) after precipitation with 20 mL acetone. The ligand-free UCNs were re-dispersed in 10 mg mL^{-1} branched PEI solution (Mw = 25,000) by sonication. After stirring for 24 h at room temperature, the products (PEI-UCNs) were collected by centrifugation (44,720 ×g for 10 min), washed with water for several time, and re-dispersed in water.

2.4) Preparation of HCy5/Cy7-UCNs (UCN) nanoplatform

The cyanine fluorophores HCy5 and Cy7 were loaded on the surface of PEG/PEI-UCNs by hydrophobic interaction following the literature published before with some modifications.[7] Briefly, polymer PEG_{5000}-COOH (100 mg) were dissolved in water (5 mL) containing EDC (47.9 mg, 0.25 mmol) and NHS (57.5 mg, 0.5 mmol) to form the active succinimidyl ester for 30 min. Then the mixture was added slowly to 5 mL PEI-UCNs (4 mg mL^{-1}) in PBS (0.1 M, pH 7.4) with 30 min sonication.[8] After vigorous magnetic stirring overnight, the products were purified by centrifugation (36,200 ×g, 10 min) and re-dispersed in 2 mL PBS solution (10 mg mL^{-1}). Then the mixture of Cy7 (10 mg) and HCy5 (2 mg) in dimethyl sulfoxide (DMSO) were added drop-wisely to the PEG/PEI-UCNs solutions in a 10 mL vial under sonication for 30 min. Finally, the mixture was stirred vigorously for overnight at room temperature, and the HCy5/Cy7-UCNs (UCN) nanoplatform was collected by centrifugation (36,200 ×g, 10 min) followed by several times washing to remove excess reactant. The loading amounts of Cy7 was determined based on its specific absorption peak at 780 nm, and the embedded HCy5 molecule was extracted by DMSO for further HPLC analysis based on its specific absorbance at 385 nm. The fluorescence quantum yields (QY) of Cy7 before (5.10 ± 0.61 %) and after (3.89 ± 0.34 %) loading on the UCNs nanoplatform were determined by following the reported methods previously.[9]

3. Prepare the stock solution of various radical species

The different stock solutions of these radical species were prepared by following the reported methods published before.[10]

3.1) $OONO^-$: The peroxynitrite ($ONOO^-$) stock solution was prepared by adding three kinds of precursor solutions, including the mixture of hydrogen peroxide (0.7 M, 1.5 mL) in hydrochloric acid (0.6 M, 1.5 mL), sodium nitrite solution (0.6 M, 3 mL) and sodium hydroxide solution (1.5 M, 3 mL) simultaneously. The resulting solution was stored at -20 ºC for further use. The concentration of the

$OONO^-$ solution was determined by measuring the absorbance at 302 nm with a molar extinction coefficient of 1670 M^{-1} cm^{-1} in 0.1 M NaOH.

3.2) ClO^-: The hypochlorite (ClO^-) stock solution was prepared by dissolving appropriate commercial NaClO solution (4-5%) in 10 mL deionized water and the concentration of the ClO^- was determined by measuring the absorbance at 209 nm with a molar extinction coefficient of 350 M^{-1} cm^{-1}.

3.3) H_2O_2: The 3% H_2O_2 stock solution was purchased from Sigma-Aldrich, which can be diluted accordingly to the desired concentration before using.

3.4) $O_2^{-\bullet}$: The stock solution of superoxide radical anion ($O_2^{-\bullet}$) was prepared based on reported methods by dissolving commercial available solid potassium superoxide (KO_2) in DMSO, or created by the enzymatic reaction of xanthine/xanthine oxidase mixture (XA/XO; 6.0 μM/3 mU) at room temperature for 5 min.[11-14] The concentration of $O_2^{-\bullet}$ was determined by measuring the absorbance at 256 nm with a molar extinction coefficient of 2686 M^{-1} cm^{-1} in 1 mM NaOH.

3.5) $^{\bullet}OH$: The $^{\bullet}OH$ stock solution (1 mM) was generated in the Fenton reaction from Iron(II) chloride ($FeCl_2$) and hydrogen peroxide (H_2O_2) under the following equation (1). Briefly, the $FeCl_2$ was dissolved in water (2 mM) and added quickly to the equivalent H_2O_2 solution (1 mM) for further use.

$$Fe^{2+} + H_2O_2 \rightarrow Fe^{3+} + {}^{\bullet}OH + OH^- \qquad (1)$$

3.6) $ROO^{\bullet}$: The peroxyl radicals ($ROO^{\bullet}$) stock solution (0.1 mM) of was prepared by diluting 0.137 mL commercial tert-Butyl hydroperoxide (t-BuOOH, 70%) in 10 mL water.

3.7) NO: The stock solution of nitric oxide (NO, 1 mM) was generated from sodium nitroferricyanide-(III) dehydrate ($Na_2[Fe(CN)_5NO]\cdot 2H_2O$, 3.0 mg) in 10 mL water.

4. MSOT imaging experimental protocol and parameters

Phantom *in vitro* and *in vivo* optoacoustic (OA) signal were performed using commercial iThera MSOT imaging system.[8] Typically, the optical excitation was provided by an optical parametric oscillator (OPO) with a tunable NIR wavelength which ranges from 680 nm to 980 nm that is in turn pumped by a Q-switched Nd:YAG laser with an average pulse duration of about 10 ns and repetition rate of 10 Hz. The OA signals were recorded by using a 128-element concave transducer array spanning a circular arc of 270°. This transducer array has a central frequency of 5 MHz, which is used to provide a transverse spatial resolution in the range of 150-200 mm. During the process of MSOT image acquisition, the *in vitro* OA signals of UCNs probe in buffers or in living cells were performed by encapsulating the solutions into an OA phantom containing two-channel polyurethane cylindrical, one for holding the control medium (working buffer) and the other for holding UCNs probe in the absence and presence of ROS/RNS treatment. The OA signals were recorded using a 128-element concave transducer array spanning a circular arc of 270° with the optimal excitation wavelength from 680 nm to 980 nm. Moreover, during the process of *in vivo* imaging, the ultrasound gel was daubed uniformly on the skin of nude mouse, and the OA signals measurements were performed subsequently in temperature-controlled water (34 °C) for acoustic coupling. The whole body MSOT imaging was operated under conditions of 0.3 mm step distance along the long axis of animals and 10 repeat pulse per position in designed time points, and

the averaged OA signal intensity in the region of interest (ROI) of liver was measured by the iThera MSOT imaging software. The ratiometric OA signals of nanoprobe (UCN) for ROS and RNS tracking at 680 nm and 800 nm were performed based on the following formula (2):

$$(\Delta OA_{680}+\Delta OA_{800})/OA_{800} = [(OA_1 - OA_0)_{680} + (OA_1 - OA_0)_{800}) / OA_{800}] \quad (2)$$

Where OA_0 and OA_1 are the optoacoustic signals of UCNs at 680 nm and 800 nm in the absence and presence of free radicals generation upon different drugs administration, and OA_{800} is the OA signals of UCN at 800 nm after various drugs treatment.

5. Multiple radical species detection in buffers

For the *in vitro* radicals sensing, different concentration of multiple free radicals including $OONO^-$, ClO^-, $O_2^{-\bullet}$, $^{\bullet}OH$, $ROO^{\bullet}$, NO and H_2O_2 were added to 1 mL UCNs nanoprobe solution (1 mg mL^{-1}) in PBS (pH = 7.4), respectively. The mixture solutions were kept at ambient temperature for 5 min followed by further UV-vis absorption and fluorescence emission spectra tests in 1 cm cuvette, and their UCL spectra were further recorded upon continues 980 nm NIR laser irradiation (2 W cm^{-2}). Moreover, in order to further investigate the different penetration depth between MSOT and UCL imaging system, the pork tissue (adipose tissue) at different thickness (2-25 mm) was utilized to mimic clinical skin by wrapping up the cylindrical holders containing UCN solution (1 mg mL^{-1}), and the OA signals from 680 nm to 980 nm were recorded by iThera MSOT imaging system. In addition, the UCL signals at 660 nm and 800 nm were separately determined based on IVIS Lumina II imaging system with specific filters (E_m: 640/50 nm and E_m: 790/30 nm) upon 980 nm NIR laser irradiation (10 W cm^{-2}). The lifetime decay curves of UCNs nanoprobe at 800 nm was determined on the lifetime spectrometer (FSP920), and the luminescence resonance energy transfer (LRET) efficiency (E) from the donor (UCNs) to acceptor (Cy7) at 800 nm could be calculated based on the following formula (3):

$$E = 1 - \tau_{DA} / \tau_D \quad (3)$$

Where τ_{DA} and τ_D are the lifetime of UCNs in the presence and absence of fluorophore (*e.g.*, Cy7) loading, respectively.

Moreover, quantitative mathematical deconvolution technique was utilized to analyze the MSOT imaging results and to further differentiate the response of ROS and RNS orthogonally in their mixture based on the OA signals at 680 nm and 800 nm by following the reported methods previously.[15-17] In our experiments, the OA spectra upon treatment of ROS-RNS combination were extracted by the iThera MSOT imaging software and specifically separated to dual peaks at 680 nm and 800 nm respectively by utilizing typical mathematical deconvolution process, as shown in supplementary Fig. 29. The quantitative analysis of ROS and RNS in the mixture could be achieved based on the following established procedures.

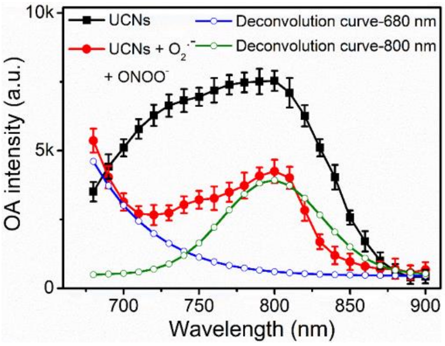

Supplementary Fig. 29. OA spectra of UCN nanoprobe (1 mg mL^{-1}) in the absence (black) and presence (red) of both ROS ($O_2^{\bullet-}$, 20 μM) and RNS ($ONOO^-$, 20 μM) in their mixture, and processed with mathematical deconvolution method by fitting the spectrum with Gaussian curves at 680 nm (blue) and 800 nm (green) respectively (R^2=0.969).

Normally, the spectral deconvoluted OA signals at 680 nm and 800 nm are directly proportional to the concentration of regenerated Cy5 (from HCy5) and Cy7 molecules upon ROS and RNS treatment, which could be denoted as the sum of each component in the mixture (or a 2 × 2 matrix equation (4)):

$$\begin{cases} OA_{680} = \varepsilon_a \cdot C_1 + \varepsilon_b \cdot C_2 \\ OA_{800} = \varepsilon_c \cdot C_1 + \varepsilon_d \cdot C_2 \end{cases} \quad \text{or} \quad \begin{bmatrix} OA_{680} \\ OA_{800} \end{bmatrix} = \begin{bmatrix} \varepsilon_a & \varepsilon_b \\ \varepsilon_c & \varepsilon_d \end{bmatrix} \begin{bmatrix} C_1 \\ C_2 \end{bmatrix} \tag{4}$$

where ε_a and ε_b are the proportionality constants of OA signals at 680 nm for the regenerated Cy5 (from HCy5); ε_c and ε_d are the proportionality constants of OA signals at 800 nm for Cy7 respectively; C_1 and C_2 indicate the concentration of fluorophores accordingly. The values of these proportionality constants could be obtained based on the slopes of standard curves (*e.g.,* $[OA_{680}]$ *vs* C_1, $[OA_{800}]$ *vs* C_1, $[OA_{680}]$ *vs* C_2, $[OA_{800}]$ *vs* C_2) at different concentrations of purified agents (Cy5 and Cy7) respectively. Moreover, considering the proportional relationship of ROS and RNS with the concentration variations of ROS-regenerated Cy5 (from HCy5) and RNS-degraded Cy7 molecules, the amounts of ROS and RNS could be further achieved by the following equation (5) :

$$\begin{cases} \Delta C_1 = k_1 \cdot [ROS] \\ \Delta C_2 = k_2 \cdot [RNS] \end{cases} \quad \text{or} \quad \begin{bmatrix} \Delta C_1 \\ \Delta C_2 \end{bmatrix} = \begin{bmatrix} k_1 \\ k_2 \end{bmatrix} \begin{bmatrix} [ROS] \\ [RNS] \end{bmatrix} \tag{5}$$

where ΔC_1 and ΔC_2 are the variations of regenerated Cy5 and degraded Cy7 concentration in the mixture before (C^0) and after (C') ROS and RNS treatment ($\Delta C_1 = C_1' - C_1^0$; $\Delta C_2 = C_2' - C_2^0$); k_1 and k_2 indicate the slops for standard curves (*e.g.,* ΔC_1 *vs* [ROS]; ΔC_2 *vs* [RNS]) at 680 nm and 800 nm upon different concentration of ROS and RNS treatment. Therefore, the determination of ROS and RNS could be obtained based on the following formula (6):

$$\begin{bmatrix} [ROS] \\ [RNS] \end{bmatrix} = \begin{bmatrix} k_1 \\ k_2 \end{bmatrix}^{-1} \begin{bmatrix} \varepsilon_a & \varepsilon_b \\ \varepsilon_c & \varepsilon_d \end{bmatrix}^{-1} \begin{bmatrix} \Delta OA_{680} \\ \Delta OA_{800} \end{bmatrix} \tag{6}$$

where ΔOA_{680} and ΔOA_{800} are the OA signals variations in the absence (OA_0) and presence (OA_1) of ROS and RNS treatment ($\Delta OA_{680} = (OA_1\text{-}OA_0)_{680}$; $\Delta OA_{800} = (OA_1\text{-}OA_0)_{800}$). By following these established procedures, the amounts of ROS and RNS in the mixtures and their respective contribution

to the OA signals changes at 680 nm and 800 nm could be orthogonally inferred accordingly.

6. Endogenous radical species monitoring in living cells

The murine macrophages RAW264.7 cell was purchased from American-type culture collection (ATCC) and cultured in high-glucose DMEM containing 10% fetal bovine serum (FBS), penicillin (100 I. U. mL^{-1}) and streptomycin (100 μg mL^{-1}) in a humidified incubator with 5% CO_2 at 37 °C. Firstly, the RAW264.7 cells were seeded in confocal dish overnight at a density of 1×10^5 cells per milliliter DMEM. For the endogenous radical species generation and imaging studies, the excessive $O_2^{\bullet-}$ in macrophage cells was activated by pretreating with phorbol 12-myristate 13-acetate (PMA) (200 ng mL^{-1}) for 1 h to generate oxidative stress,[18] and the excessive $ONOO^-$ in RAW264.7 cells was produced by stimulating with lipopolysaccharide (LPS, 1 μg mL^{-1}) and interferon-γ (INF-γ, 50 ng mL^{-1}) for 4 h followed by PMA (10 nM) treatment for 0.5 h to induce nitrosative stress.[10] The generation of ROS (*e.g.,* $O_2^{\bullet-}$) and RNS (*e.g.,* $ONOO^-$) were further confirmed by the standard MitoSOX mitochondrial superoxide indicator (red fluorescence) and Cell Meter™ fluorimetric intracellular peroxynitrite indicator (green fluorescence) respectively by following the manufacturer's protocols.[19, 20] To identify the specificity of these two radicals monitoring, the cells were pre-treated with Mn(III) tetrakis (4-benzoic acid) porphyrin (MnTBAP, 100 μM) as $O_2^{\bullet-}$ scavenger and mercaptoethyl guanidine (MEG, 100 μM) as $ONOO^-$ scavenger for 1 h before the oxidative or nitrosative stress generation upon PMA and LPS/IFN-γ/PMA stimulation, respectively. After refreshing the medium, the RAW264.7 cells were further incubated with UCN (100 μg mL^{-1}) for 4 h in confocal dish with 1 mL DMEM, and the UCL cellular imaging was performed in Nikon fluorescence microcopy upon 980 nm NIR light excitation (5 W cm^{-2}).

Moreover, the MSOT images in living cells were also collected by following the methods described previously.[8, 21] Briefly, the UCNs probe was incubated with the RAW264.7 macrophage cells after PMA ($O_2^{\bullet-}$ generation) and LPS/IFN-γ/PMA ($ONOO^-$ production) stimulation and collected in 1 mL DMEM (1×10^6 cells mL^{-1}). Then these cell samples in different groups were encapsulated into an OA phantom respectively containing two-channel polyurethane cylindrical, one for holding the control medium (DMEM) and the other for holding cell samples with UCNs probe treatment in different groups. The MSOT images and OA signals were finally recorded using a 128-element concave transducer array spanning a circular arc of 270° with the optimal excitation wavelength at 680 nm to 980 nm.

7. Examination of mitochondria dysfunction in living cells

In order to explore the potential mechanism of ROS/RNS-induced pathology in living cells, the membrane-permeable lipophilic cationic fluorochrome JC-1 was utilized to examine the mitochondrial membrane potential ($\Delta\psi_m$) in RAW264.7 cells by flow cytometry (FCM) analysis.[22] Briefly, the cells were firstly treated with the PMA ($O_2^{\bullet-}$ generation), LPS/IFN-γ/PMA ($ONOO^-$ production) and their specific scavengers by following the methods described previously.[10,18] Then the cells were collected and suspended in pre-warmed working buffer (1×10^6 cells mL^{-1}) containing JC-1 (2 μM) and incubated at 37 °C for 15 min. Subsequently, the cells were further washed by assay buffer twice and directly analyzed by BD LSR Fortessa™ X-20 flow cytometer with ten thousand events for each sample (monomer at green channel: E_x: 488 nm, E_m: 530/50 nm; aggregator at red channel: E_x: 561 nm, E_m: 610/75 nm).

The cytotoxicity of UCN was also determined in living RAW264.7 cells by using the standard cell viability test methods.[23] Briefly, the cells were seeded in a 96-well plate (1×10^4 cells per well in 100 μL DMEM) and cultured for 24 hours at 37 °C, and further incubated with UCN at different concentrations for 24 h. After removing the medium, the cell viabilities were evaluated by *in vitro* toxicology assay kit

(TOX8, resazurin based) by following the manufacturer's protocols. Each experiment was repeated three times and the average values were used for cytotoxicity analysis.

8. MSOT and UCL imaging of multiple radical species *in vivo*

All animal experimental procedures were performed in accordance with the protocol approved by the Institutional Animal Care and Use Committee of Soochow University. The Balb/c nude mice (~ 6-8 weeks old) were purchased from Shanghai Laboratories Animal Center in China. To study the bio-distribution of prepared nanoprobe, the nude mice were intravenously injected with UCNs (5 mg mL^{-1} in 100 μL saline) and were further sacrificed at different time points after UCNs administration. Different organs including heart, liver, spleen, lung, kidney, stomach and intestines were collected and washed with PBS (pH = 7.4) for three times, and the UCL signals of UCN were recorded based on the IVIS Lumina II imaging system upon NIR light irradiation (E_x: 980 nm, E_m: 790/30 nm).

In order to further monitor the dynamic processes of radical oxidative or nitrosative stress in the inflammation model *in vivo*, the mice were fasted overnight and intraperitoneal (*i.p.*) treated with sterilized saline solutions containing different modeling drugs including lipopolysaccharide (LPS, 20 mg Kg^{-1}), acetaminophenol (APAP, 300 mg Kg^{-1}), isoniazid (INH, 200 mg Kg^{-1}) and tacrine (THA, 30 mg Kg^{-1}), respectively (n = 5). For redox species scavenging studies, the animals were pre-treated with N-acetyl-cysteine (NAC, 200 mg Kg^{-1}) as scavenger by *i.p.* injection at one hour before drugs stimulation as indicated in previous reports. Fifteen minutes after drugs treatment, the UCN (5 mg mL^{-1} in 100 μL saline) were injected intravenously (*i.v.*) and the mice were then anesthetized with 3% isoflurane for UCL imaging at 660 nm and 800 nm on the IVIS Lumina II animal imaging system with specific filters (E_m: 640/50 nm and E_m: 790/30 nm) upon 980 nm NIR light irradiation (10 W cm^{-2}). The real-time MSOT imaging was also performed by whole body screening from 680 nm to 980 nm after drugs and UCN administration at different time points with 10 min interval. To dynamic profile the variations of radical oxidative and nitrosative stress simultaneously *in vivo*, the time-resolved MSOT signals at 680 nm and 800 nm in mouse liver with pseudo-color processing were collected upon diverse hepatotoxins injection, and the ROI intensity was recorded on the tomographic anatomical image of liver cross-section in iThera MSOT imaging software.

9. Histological and immunohistochemical analysis *in vivo*

To evaluate the tissue damage and inflammatory response by pathological sections, the animals were euthanized at 60 min and 180 min upon UCN, APAP, INH and THA treatment as described above. The various organs including liver, heart, spleen, lung and kidney were resected and placed into 4% formalin solutions overnight at 4 °C, and all tissues were embedded in paraffin followed by 10 μm sectioning. For the histological studies, these organ tissues were stained by hematoxylin and eosin (H&E) under standard protocols.[24] For the immunohistochemical staining in liver samples, the tissues were processed for 4-hydroxynonenal (4-HNE) and 3-nitrotyrosine staining based on the anti-4-HNE primary antibody and anti-3-nitrotyrosine primary antibody (Abcam) at 1:500 dilution by following the manufacturer's methods.[25, 26] All images were acquired using an Olympus IX53 inverted fluorescence microscope equipped with a Nuance (CRi Inc.) hyperspectral camera capable of bright field full-color imaging.

10. Metabolic mechanism and inflammation procession exploration *in vivo*

In order to examine the metabolic mechanism and inflammation procession during the drug induced liver injury (DILI), the mice were treated with UCN, APAP, INH and THA by following the same

protocols above, and further sacrificed to collect their livers at designed time points. Frozen liver tissues were thawed and homogenized in ice-cold PBS using a tissue homogenizer (Omni) for 10 min (1,000 ×g, 4 °C) and the homogenates were collected for the assays of several typically hepatic biomarkers, including 5'-diphospho-glucuronosyltransferases (UGTs), interleukin-6 (IL-6) and interleukin-10 (IL-10), respectively ($n = 5$). Liver microsomes in the endoplasmic reticulum of hepatic cells were also isolated from liver tissues by utilizing the classical methods reported previously to determine the amounts of the cytochrome P450 (CYP450).[27] Both of these specific biomarkers were determined by enzyme-linked immunosorbent assay (ELISA) kit according to the manufacturer's procedures, and the protein content in liver tissues was determined by bicinchoninic acid (BCA) method based on the standard protocols.[28] Moreover, to determine the hepatotoxicity upon diverse drugs treatment, the mice were anesthetized and the blood was collected from the vena cava at different time points , and the serum was separated immediately to measure aspartate aminotransferase (AST) and alanine aminotransferase (ALT) by following the standard protocols($n = 5$).[29]

11. Statistical analysis

Quantitative data are represented as mean ± standard deviations (SD) unless specifically described. All of the measurements are taken from distinct samples, and the statistical significance are assessed by a Student's t-test (heteroscedastic, two-sided): *$p < 0.05$, **$p < 0.01$, ***$p < 0.001$).

Supplementary References

1. Tsai, Y.C. *et al.* Targeted Delivery of Functionalized Upconversion Nanoparticles for Externally Triggered Photothermal/Photodynamic Therapies of Brain Glioblastoma. *Theranostics* **8**, 1435-1448 (2018).
2. Chen, Z. *et al.* Cancer Cell Membrane-Biomimetic Nanoparticles for Homologous-Targeting Dual-Modal Imaging and Photothermal Therapy. *Acs Nano* **10**, 10049-10057 (2016).
3. Oushiki, D. *et al.* Development and application of a near-infrared fluorescence probe for oxidative stress based on differential reactivity of linked cyanine dyes. *J. Am. Chem. Soc.* **132**, 2795-2801 (2010).
4. Kundu, K. *et al.* Hydrocyanines: a class of fluorescent sensors that can image reactive oxygen species in cell culture, tissue, and in vivo. *Angew. Chem. Int. Ed.* **48**, 299-303 (2009).
5. Wang, F., Deng, R. & Liu, X. Preparation of core-shell NaGdF 4 nanoparticles doped with luminescent lanthanide ions to be used as upconversion-based probes. *Nat. Protoc.* **9**, 1634 (2014).
6. Bogdan, N., Vetrone, F., Ozin, G.A. & Capobianco, J.A. Synthesis of ligand-free colloidally stable water dispersible brightly luminescent lanthanide-doped upconverting nanoparticles. *Nano Lett.* **11**, 835-840 (2011).
7. Sedlmeier, A. & Gorris, H.H. Surface modification and characterization of photon-upconverting nanoparticles for bioanalytical applications. *Chem. Soc. Rev.* **44**, 1526-1560 (2015).
8. Ai, X. *et al.* In vivo covalent cross-linking of photon-converted rare-earth nanostructures for tumour localization and theranostics. *Nat. Commun.* **7**, 10432 (2016).
9. Wurth, C., Grabolle, M., Pauli, J., Spieles, M. & Resch-Genger, U. Relative and absolute determination of fluorescence quantum yields of transparent samples. *Nat. Protoc.* **8**, 1535-1550 (2013).
10. Jia, X. *et al.* FRET-based mito-specific fluorescent probe for ratiometric detection and imaging of endogenous peroxynitrite: dyad of Cy3 and Cy5. *J. Am. Chem. Soc.* **138**, 10778-10781 (2016).
11. Zhang, W. *et al.* Dynamic and reversible fluorescence imaging of superoxide anion fluctuations in live cells and in vivo. *J. Am. Chem. Soc.* **135**, 14956-14959 (2013).
12. Wang, Y. *et al.* Associated Detection of Superoxide Anion and Mercury(II) under Chronic Mercury Exposure in Cells and Mice Models via a Three-Channel Fluorescent Probe. *Anal. Chem.* **90**, 9769-9778 (2018).
13. Georgiou, C.D., Papapostolou, I., Sun, H. & McKay, C.P. Superoxide radical assays and applications in Mars-like Atacama soils. *J. Geophys. Res.-Biogeosci.* **112**, 6 (2007).
14. Samuel, E.L.G. *et al.* Highly efficient conversion of superoxide to oxygen using hydrophilic carbon clusters. *Proc. Natl. Acad. Sci. U. S. A.* **112**, 2343-2348 (2015).
15. Antonov, L. & Nedeltcheva, D. Resolution of overlapping UV-Vis absorption bands and quantitative analysis. *Chem. Soc. Rev.* **29**, 217-227 (2000).
16. Domenici, V. *et al.* Extraction of Pigment Information from Near-UV Vis Absorption Spectra of Extra Virgin Olive Oils. *J. Agric. Food. Chem.* **62**, 9317-9325 (2014).
17. Karabudak, E. *et al.* Simultaneous Identification of Spectral Properties and Sizes of Multiple Particles in Solution with Subnanometer Resolution. *Angew. Chem.-Int. Edit.* **55**, 11770-11774 (2016).
18. Hu, J.J. *et al.* Fluorescent probe HKSOX-1 for imaging and detection of endogenous superoxide in live cells and in vivo. *J. Am. Chem. Soc.* **137**, 6837-6843 (2015).
19. https://www.thermofisher.com/order/catalog/product/M36008.
20. https://www.abcam.com/peroxynitrite-assay-kit-cell-based-ab233468.html.

21. Jiang, X. *et al.* Ultrasmall Magnetic CuFeSe2 Ternary Nanocrystals for Multimodal Imaging Guided Photothermal Therapy of Cancer. *Acs Nano* **11**, 5633-5645 (2017).
22. https://assets.thermofisher.com/TFS-Assets/LSG/manuals/mp34152.pdf.
23. Ai, X. *et al.* Remote Regulation of Membrane Channel Activity by Site-Specific Localization of Lanthanide-Doped Upconversion Nanocrystals. *Angew. Chem. Int. Ed.* **56**, 3031-3035 (2017).
24. http://www.aladdin-e.com/up_files/docs/Hematoxylin%20&%20Eosin%20Staining%20Protocol.pdf.
25. Shuhendler, A.J., Pu, K., Cui, L., Uetrecht, J.P. & Rao, J. Real-time imaging of oxidative and nitrosative stress in the liver of live animals for drug-toxicity testing. *Nat. Biotechnol.* **32**, 373 (2014).
26. Abusarah, J. *et al.* An overview of the role of lipid peroxidation-derived 4-hydroxynonenal in osteoarthritis. *Inflammation Res.* **66**, 637-651 (2017).
27. Kamath, S. & Rubin, E. Interaction of calcium with microsomes: a modified method for the rapid isolation of rat liver microsomes. *Biochem. Biophys. Res. Commun.* **49**, 52-59 (1972).
28. https://www.thermofisher.com/sg/en/home/life-science.
29. http://www.abcam.cn/Alanine-Transaminase-Activity-Assay-Kit-ColorimetricFluorometric-ab105134. html.
