# Supplementary material for: Multispectral optoacoustic imaging of dynamic redox correlation and pathophysiological progression utilizing upconversion nanoprobes
Source: Nat Commun. 2019 Mar 6;10:1087. doi: 10.1038/s41467-019-09001-7 (PMC6403272; doi:10.1038/s41467-019-09001-7)
Supplement: Supplementary file 3 — Reporting Summary [file 41467_2019_9001_MOESM3_ESM.pdf]

nature research

Corresponding author(s): Bengang Xing

Last updated by author(s): Jan 26, 2019

Reporting Summary

Nature Research wishes to improve the reproducibility of the work that we publish. This form provides structure for consistency and transparency in reporting. For further information on Nature Research policies, see Authors & Referees and the Editorial Policy Checklist.

Statistics

For all statistical analyses, confirm that the following items are present in the figure legend, table legend, main text, or Methods section.

n/a	Confirmed	
☐	☒	The exact sample size (*n*) for each experimental group/condition, given as a discrete number and unit of measurement
☐	☒	A statement on whether measurements were taken from distinct samples or whether the same sample was measured repeatedly
☐	☒	The statistical test(s) used AND whether they are one- or two-sided<br>*Only common tests should be described solely by name; describe more complex techniques in the Methods section.*
☐	☒	A description of all covariates tested
☐	☒	A description of any assumptions or corrections, such as tests of normality and adjustment for multiple comparisons
☐	☒	A full description of the statistical parameters including central tendency (e.g. means) or other basic estimates (e.g. regression coefficient) AND variation (e.g. standard deviation) or associated estimates of uncertainty (e.g. confidence intervals)
☐	☒	For null hypothesis testing, the test statistic (e.g. *F*, *t*, *r*) with confidence intervals, effect sizes, degrees of freedom and *P* value noted<br>*Give P values as exact values whenever suitable.*
☐	☒	For Bayesian analysis, information on the choice of priors and Markov chain Monte Carlo settings
☐	☒	For hierarchical and complex designs, identification of the appropriate level for tests and full reporting of outcomes
☐	☒	Estimates of effect sizes (e.g. Cohen's *d*, Pearson's *r*), indicating how they were calculated

Our web collection on statistics for biologists contains articles on many of the points above.

Software and code

Policy information about availability of computer code

Data collection	No software was used
Data analysis	No software was used

For manuscripts utilizing custom algorithms or software that are central to the research but not yet described in published literature, software must be made available to editors/reviewers. We strongly encourage code deposition in a community repository (e.g. GitHub). See the Nature Research guidelines for submitting code & software for further information.

Data

Policy information about availability of data

All manuscripts must include a data availability statement. This statement should provide the following information, where applicable:

- Accession codes, unique identifiers, or web links for publicly available datasets
- A list of figures that have associated raw data
- A description of any restrictions on data availability

The authors declare that all the data supporting the findings of this study are available from the authors on reasonable request.

Field-specific reporting

Please select the one below that is the best fit for your research. If you are not sure, read the appropriate sections before making your selection.

☒ Life sciences ☐ Behavioural & social sciences ☐ Ecological, evolutionary & environmental sciences

For a reference copy of the document with all sections, see nature.com/documents/nr-reporting-summary-flat.pdf

Life sciences study design

All studies must disclose on these points even when the disclosure is negative.

Sample size	The sample size in our studies (n=5) was assessed based on the statistical significance of a Student's t-test (heteroscedastic, two-sided).
Data exclusions	No data were excluded from the analyses in this study.
Replication	We repeated each measurement in our studies for at least 5 times, and all attempts at replication were successful.
Randomization	The samples (e.g., living mice) were allocated to different experimental groups randomly.
Blinding	We confirm that the investigators were blinded to group allocation during date collection and analysis.

Reporting for specific materials, systems and methods

We require information from authors about some types of materials, experimental systems and methods used in many studies. Here, indicate whether each material, system or method listed is relevant to your study. If you are not sure if a list item applies to your research, read the appropriate section before selecting a response.

Materials & experimental systems

n/a	Involved in the study	
☐	☒	Antibodies
☐	☒	Eukaryotic cell lines
☒	☐	Palaeontology
☐	☒	Animals and other organisms
☒	☐	Human research participants
☒	☐	Clinical data

Methods

n/a	Involved in the study	
☒	☐	ChIP-seq
☐	☒	Flow cytometry
☒	☐	MRI-based neuroimaging

Antibodies

Antibodies used	Anti-4 Hydroxynonenal antibody (Abcam, ab46545, USA); Anti-3-Nitrotyrosine antibody (Abcam, ab61392, USA)
Validation	The validation of each primary antibody on the manufacturer's website (Abcam):<br>For Anti-4 Hydroxynonenal antibody: https://www.abcam.com/top-306.3999938964844<br>For Anti-3-Nitrotyrosine antibody: https://www.abcam.com/3-nitrotyrosine-antibody-39b6-ab61392.html

Eukaryotic cell lines

Policy information about cell lines

Cell line source(s)	American-type culture collection (ATCC, cat. no. TIB-71).
Authentication	None of the cell lines used were authenticated.
Mycoplasma contamination	We confirm that all cell lines tested negative for mycoplasma contamination.
Commonly misidentified lines (See ICLAC register)	The RAW264.3 macrophage cell line we used in this study was not listed by ICLAC as misidentified cell line.

Animals and other organisms

Policy information about studies involving animals; ARRIVE guidelines recommended for reporting animal research

Laboratory animals	Balb/c nude mice, Female, 6-8 weeks old.
Wild animals	The study did not involve wild animals.
Field-collected samples	The study did not involve samples collected from the field.
Ethics oversight	All animal experimental procedures were performed in accordance with the protocols approved by the Institutional Animal Care and Use Committee of Soochow University in China.

Note that full information on the approval of the study protocol must also be provided in the manuscript.

Flow Cytometry

Plots

Confirm that:

☒ The axis labels state the marker and fluorochrome used (e.g. CD4-FITC).

☒ The axis scales are clearly visible. Include numbers along axes only for bottom left plot of group (a 'group' is an analysis of identical markers).

☒ All plots are contour plots with outliers or pseudocolor plots.

☒ A numerical value for number of cells or percentage (with statistics) is provided.

Methodology

Sample preparation	The radicals treated RAW264.7 murine macrophage cells were detached by 0.25% trypsin and collected by centrifuge.
Instrument	Flow cytometry (FCM) analysis was performed using a BD LSRFortessa™ X-20 cell analyzer (USA).
Software	The FCM data were analyzed based on the professional and commercial software FlowJo 7.6.1 (USA).
Cell population abundance	The FCM data present single cell population within post-sort fractions, suggesting the great purity of cell samples for FCM analysis.
Gating strategy	The gate of all relevant FCM experiments was defined based on the cells in control group (un-treated RAW264.7 cells incubate with JC-1 indicator), which indicating the "positive" and "negative" boundaries of JC-1 monomer (green, FITC-H in X-axis) at 10^3 and JC-1 aggregator (red, PE-H in Y-axis) at 10^3, respectively. The preliminary FSC/SSC gates of cell population were started from 10^4 for all the samples in our studies.

☒ Tick this box to confirm that a figure exemplifying the gating strategy is provided in the Supplementary Information.
